# Dr. Otto Heinrich Warburg—Survivor of Ethical Storms

**DOI:** 10.5041/RMMJ.10183

**Published:** 2015-01-29

**Authors:** George M. Weisz

**Affiliations:** Senior Lecturer, School of Humanities (Program in History of Medicine), University of New South Wales, Sydney, Australia, and University of New England, Armidale, New South Wales, Australia

**Keywords:** Nobel, Otto Heinrich Warburg, Warburg effect

## Abstract

Otto Heinrich Warburg (1883–1970; not to be confused with the Zionist of the same name) was a member of an illustrious Jewish family, known for some five centuries. From humble beginnings, the family became prominent in the world for their contributions to all aspects of society. The son of a German mother and a Jewish (converted) father, Otto H. Warburg became a major contributor to medical science in the field of cancer research. Considered for Nobel Prize more than once, he finally received it in 1931 for his discovery of the nature and mode of action of the cellular respiratory enzyme. Warburg’s personality was controversial: he was intolerant of opposing scientific views yet tolerant toward Nazi abuses. Accused of collaboration under the Nazi regime, Otto H. Warburg was nevertheless readmitted to the global scientific community after World War II. His contribution to cancer research remains influential to this day and has been superseded by discoveries that have built upon his work.

## INTRODUCTION

The passage of time, I believe, will increase Warburg’s status as a scientist.

(Hans Krebs, Nobel Laureate^1^)

**Figure 1.**
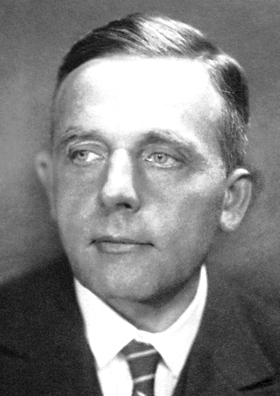
**Otto Heinrich Warburg.**

Who was Otto Heinrich Warburg (Figure 1)? He was an “eccentric genius … who devoted his entire life to the pursuit of science.”^1^ His private life was controversial, he kept a distance from his extended family, and he had no contact with the Jewish community. He could be sarcastic and vindictive, as well as polite and generous, albeit quite demanding of his assistants. He led a solitary life, cohabitated with his companion, a friend who eventually became an organizer of the Kaiser Wilhelm Institute.

## FAMILY BACKGROUND

The del Banco (Delbanco) family were Jewish refugees from Portugal, arriving in Venice in the 1500s. Upon establishment of the Jewish Ghetto in Venice, the family moved to Germany. In 1559, they settled in the city of Warburg (Westphalia, bordering Holland). During the seventeenth century, Jews were encouraged by the Prince Bishop to settle in Warburg, where Simon of Cassel, the representative of the Jewish community, obtained certain rights to work, provided they adopted the city’s name as their family name—hence the family name, Warburg. Subsequently, Simon Warburg’s grandson, Jacob Samuel, refused the demand for conversion, and the family resettled in Altona, near Hamburg, but under the Danish Crown.

The Warburg family flourished and maintained their Jewish identity, with descendants named Samuel, Isaac, Moritz, Moses, and Jacob. However, in later years, non-Jewish names such as Karl, Johann, Pius, Felix, Aby, August, Adolph, Emil, and Otto were used.^2^–^4^ As a result of the emancipation experienced in the nineteenth and twentieth centuries, the very extended family became active in some 32 occupations. The family split into two main branches: an American branch that worked primarily in the financial industry in New York, and a European branch that founded the Warburg Bank in Hamburg and London. The family was also active in the arts, building up the great “Bibliothek” in Hamburg, which was later transferred to London as the “Warburg Institute,” and establishing a large printing company.

There is no record of medical practitioners along the centuries; however, three were mentioned during the Second World War. The first mentioned was Professor Carl Warburg; he was arrested by the Gestapo, and subsequently released at the insistence of his faithful patient, King Christian of Denmark. The second was Dr Betty Warburg, a general practitioner residing in Altona, near Hamburg.^4^,^5^ The third was Otto Heinrich Warburg.

## LIFE AND SCIENTIFIC LEGACY OF OTTO H. WARBURG

The personal life and the scientific legacy of Otto H. Warburg are difficult to separate. In fact, it seems that very little could separate him from his research.

### Personal Life

Otto Heinrich Warburg was born to a Protestant mother in Freiburg im Bresgau in 1883, close to the Swiss border. His father, Emil, had converted to Protestantism as an adult, although Emil’s parents were Orthodox Jews. Emil was a distinguished Professor of Physics in Freiburg. The Warburg family had moved to Berlin upon Emil’s appointment as Head of the Physics Department in the Imperial Institute for Physics and Technology (Physikalisch-Technische Reichsanstalt).

Hence, Warburg grew up in an academic environment, inspired by his father’s excellence. In later years his early experiments on the quantum requirement of photosynthesis were conducted in his father’s laboratory.^6^

As he became older, finding a cure for cancer became Warburg’s overriding ambition. He graduated with a PhD in biochemistry from the University of Berlin in 1906, and received his MD from the University of Heidelberg in 1911. Warburg’s postgraduate studies brought him repeatedly to the Stazione Zoologica Anton Dohrn of Naples, Italy, to study urchin eggs and the respiratory process in normal and growing cells. The impact of those early studies on his future cancer research proved to be most significant. At times controversial, his work was eventually appreciated by the Nobel Committee.

In 1914, Warburg moved to the University of Berlin’s distinguished Kaiser Wilhelm Institute. Soon, he became head of the Cell Physiology Research Laboratory, established and financed by the Rockefeller Foundation. From there, over the next 50 years, Warburg would develop his many theories and publish 508 articles and five books.

His many years of research were first interrupted for four years during World War I. Warburg volunteered in the army, working first as a physician and later on in the army headquarters. He was injured and decorated with the Iron Cross, Class I. He continued to serve until mid-1918, when at the instigation of a group of academics (headed by Albert Einstein), Warburg resumed his work in cancer research.^2^–^5^

Warburg looked back on his four years of service to Germany in World War I with pride. Although he did not deny his Jewish origin, he considered himself a German patriot.^4^,^5^ His work continued and was only briefly interrupted during World War II. In the summer of 1945, the Russians confiscated his equipment; however, Warburg himself was respected. His Institute was later rebuilt in Berlin’s American zone, Dahlem.

Warburg remained a bachelor and resided in the institute with his faithful companion, Jacob Heiss, a personal friend and the secretary and manager of the Kaiser Wilhelm Institute.^2^ Warburg pursued his research until the age of 87. In 1968, he suffered a broken femur, complicated by deep vein thrombosis, and in 1970, Otto Heinrich Warburg died from a pulmonary embolism and was buried in a Christian cemetery.

### Scientific Legacy

Otto Heinrich Warburg’s research between 1911 and 1970 focused on photosynthesis and the chemistry of the enzymes in cell respiration (redox reaction; i.e. reduction and oxygenation) in normal and cancer cells.^1^,^2^

Warburg introduced technical advances in experimentation that have since become standard tools, still in use today. He improved the methodology for gas analysis in processes such as cell respiration and photosynthesis, and invented a manometer referred to as the Warburg apparatus; he also invented the spectrophotometer and developed a tissue-slicing technique for measuring cell metabolism. He discovered the cell respiratory enzymes, tried to find the energy source for cell growth, and oxygenation in normal versus cancer cells. He also introduced a theory for the “primary cause for cancer,” namely abnormal cell respiration, referred to as “the Warburg effect.”^7^

Warburg determined that the “secondary source of cancer” was exogenous irritants such as nicotine, food additives, air pollution, and exhaust from motor vehicles.^2^,^7^

His theory regarding the primary source of cancer, namely fermentation (glycolysis) under low-pressure O_2_, was only partially accepted by future researchers. Warburg’s work added to the well-known theory that genetic factors contributed to tumor growth.

It was Warburg’s discovery of “cell respiratory enzymes” in 1924 that resulted in his being considered for the Nobel Prize in 1927. However, it was awarded to someone else.^1^ Warburg was finally awarded the Nobel Prize in 1931 for his studies on cell combustion, or more precisely “oxygen transfer by enzymes.”^8^,^9^

The essence of the Warburg effect was that malignant cells, starved of oxygen, would transfer to a primitive form of fermentation as a source of energy, namely glycolysis, resulting in acidosis in the body (pH 6).^9^,^10^ Warburg found that cultured liver cancer cells require more fermentation, paralleling their degree of malignancy.^10^,^11^ He was criticized for denying the effect of genes and of viruses simply because of the “absence of evidence.”

In 1940, Warburg expressed his hope for a third nomination within two years, “when the problem of cancer will be resolved.”^2^

He continued this line of research, and in 1944 his study of “hydrogen transfer of cell enzymes” attracted a third Nobel nomination that was highly politicized. Warburg was nominated by another Nobel Laureate, Szent-Gyorgyi, for the discovery of nicotinamide (later on utilized by others in the cure of pellagra) and flavins (yellow enzymes). It has been rumored that the Nobel Committee actually did give Warburg the prize, but then rescinded it in response to Germany’s refusal to allow their nationals to accept international prizes. In any case, Warburg would not have been allowed to leave the country.^1^,^7^ This rumor relating to the 1944 prize is but one of the unresolved controversies in Warburg’s life. In any case, officially, the award went to someone else.^2^,^5^

In later years, Warburg’s theories, dormant for many years, re-emerged and were followed by several new studies.^12^–^16^ Warburg’s theories were revived in 1966 at a Nobel Laureates conference in the Bavarian city of Lindau, where he presented “The Prime Cause and Prevention of Cancer.”^9^

Warburg’s long-standing theory of altered glucose metabolism in cancer cells was eventually verified via positron emission; that theory led to the current-day use of 2-18F-2-deoxyglucose (FDG) for PET scanning of metastatic formations.^14^ Recently, a “reverse Warburg effect” has been implicated in the pathogenesis of Alzheimer’s disease.^17^

### The Nazi Controversy

Behind the scenes of Otto Heinrich Warburg’s contributions to science, and under the surface of his family relationships, lies a controversy with possible ethical implications.

Most of the extended Warburg family escaped from Nazi persecution during World War II. However, Dr Betty Warburg and her mother Gerta perished in Sobibor; a cousin, Helen, perished in Auschwitz, and another cousin, Maria, never left the Brandenburg Euthanasia Center.^18^ Otto’s three sisters survived by marrying members of Germany’s high society and converting to Christianity.

Warburg’s survival in Nazi Germany has aroused considerable controversy. The question has been raised as to why Warburg was able to remain un-molested in Berlin throughout the 12 years of the Third Reich. Warburg was the grandson of Orthodox Jews (Daniel Marcus Warburg and Ida Cohen), and the son of Emil Warburg—albeit a convert to Protestantism. Although according to Judaic Law Warburg was not considered Jewish, under the Nazis he should have been deported. Indeed, in accordance with the Nazi Racial Laws, his status would have been a “*Mischling*”—50% Jew.

In 1941, Otto Warburg’s research work was interrupted, but only for three weeks. He was dismissed from his position as Head of the Kaiser Wilhelm Institute and not permitted to teach or take up an academic position. He was then reinstated at the order of Hitler’s Chancellery (Bouhler), and Goring ordered that Warburg’s genealogy be re-assessed at 25% Jew status under the declaration, “I will decide who is a Jew.”^4^,^8^,^19^ There is anecdotal evidence of the Fuehrer’s oncophobia following removal of a laryngeal polyp. Although the rumor is unproven, it might be connected to the reassessment of Warburg’s Jewish status. Warburg’s continuing employment, despite the Civil Service Law of 1933 that barred Jews from working, could also have been due to the dependence of the Institute of Cell Research on funds awarded by the American Rockefeller and British Gradenwitz organizations—at least until the start of World War II. By then, the Kaiser Wilhelm Gesellschaft (later renamed the Max Planck Institute) had been purged of Jews, but Otto Warburg was “saved for the world” by his connections with the Reichwehr and in particular with Viktor Brack and Phillip Bouhler, both holding high-ranking positions in the SS and the government.^11^,^20^,^21^

Warburg had been quoted unofficially criticizing some issues relating to the Nazis, but in general he remained apolitical—a strict scientist. He lived in the Kaiser Wilhelm Institute, worked six days a week, and, although many other scientists left or were dismissed, Warburg remained in Berlin. He was shunned by foreign scientists for his tacit acceptance of anti-Jewish measures taken against his colleagues and the rest of the Jewish people.^1^,^8^ Warburg’s explanation for remaining in Berlin was the excellent research team that had taken him years to assemble.

Warburg’s student and biographer, Nobel Laureate Hans Krebs, wrote: “Warburg’s willingness to let his Jewish blood be diluted in this way, and thus to make a pact with the Nazis, incensed colleagues outside Germany.”^1^ Indeed, attempts by Warburg to relocate to America after the war found a cool response. Nevertheless, Warburg seemed to have continued to live undisturbed and unaffected by the “identity crisis” that perturbed his entire family after 1933 due to the lack of reciprocity and betrayal of their patriotism expressed during World War I and under the Weimar republic.^8^

After the Second World War, Warburg was readmitted into the international scientific community. By 1952, with numerous accolades accorded to him, he was often invited to lecture in Europe and the US. Also, despite international censure, his membership in the Royal Society (since 1934) was not rescinded during the war. In fact, Warburg received an honorary doctorate from Oxford University in 1965 and was invited to participate in the Lindau Conference in 1966.

Was Warburg’s behavior a cohabitation or collaboration with the Nazi regime? This question remains a puzzle open to personal interpretation. His attitude could be considered as collaboration, since Germany benefited from his publications. However, since Warburg performed no known unethical human experiments, his research results could be accepted as not being immorally obtained. Warburg’s list of publications reveals 207 articles and two books to his credit prior to the ascent of the Nazi regime in 1933. During his 12 years under the Third Reich, Warburg published another 105 scientific articles. Following the war he went on to publish another 191 articles and three books.

## OTTO HEINRICH WARBURG—A SURVIVOR

It is widely accepted that Warburg was a brilliant researcher, with innovative experimental methods, pedantic, addicted to research, demanding of his team, reclusive, self-sufficient, critical, and controversial. Nevertheless, his scientific legacy remains important to this day.

His triple sins in the Nazi ideology were his Jewish ancestry, some anti-Nazi criticism, and his probable sexual orientation. All three were hypocritically overlooked, and Warburg remained unscathed. Perhaps it is one of the numerous illogical Nazi theorizations. Or, rather, was it a reluctant recognition of a brilliant mind, even within the hated race?

This same hypocritical attitude was held by the scientific world: ostracized during World War II, Warburg was hesitantly welcomed back to its fold after the war. By the end of his life, Warburg’s “sins” had been forgiven by one and all. Nevertheless, the life of Otto Heinrich Warburg and his ability to survive make an interesting footnote to his enduring contributions to cancer research. Perhaps, like the cure for all disease, some questions will never be answered.
